# A γ-Secretase Inhibitor, but Not a γ-Secretase Modulator, Induced Defects in BDNF Axonal Trafficking and Signaling: Evidence for a Role for APP

**DOI:** 10.1371/journal.pone.0118379

**Published:** 2015-02-24

**Authors:** April M. Weissmiller, Orlangie Natera-Naranjo, Sol M. Reyna, Matthew L. Pearn, Xiaobei Zhao, Phuong Nguyen, Soan Cheng, Lawrence S. B. Goldstein, Rudolph E. Tanzi, Steven L. Wagner, William C. Mobley, Chengbiao Wu

**Affiliations:** 1 Department of Neurosciences, University of California San Diego, San Diego, California, United States of America; 2 Department of Cellular and Molecular Medicine, University of California San Diego, San Diego, California, United States of America; 3 Department of Anesthesiology, University of California San Diego, San Diego, California, United States of America; 4 V.A. San Diego Healthcare System, San Diego, California, United States of America; 5 Genetics and Aging Research Unit, Department of Neurology, Massachusetts General Hospital, Charlestown, Massachusetts, United States of America; National Center for Geriatrics and Gerontology, JAPAN

## Abstract

Clues to Alzheimer disease (AD) pathogenesis come from a variety of different sources including studies of clinical and neuropathological features, biomarkers, genomics and animal and cellular models. An important role for amyloid precursor protein (APP) and its processing has emerged and considerable interest has been directed at the hypothesis that Aβ peptides induce changes central to pathogenesis. Accordingly, molecules that reduce the levels of Aβ peptides have been discovered such as γ-secretase inhibitors (GSIs) and modulators (GSMs). GSIs and GSMs reduce Aβ levels through very different mechanisms. However, GSIs, but not GSMs, markedly increase the levels of APP CTFs that are increasingly viewed as disrupting neuronal function. Here, we evaluated the effects of GSIs and GSMs on a number of neuronal phenotypes possibly relevant to their use in treatment of AD. We report that GSI disrupted retrograde axonal trafficking of brain-derived neurotrophic factor (BDNF), suppressed BDNF-induced downstream signaling pathways and induced changes in the distribution within neuronal processes of mitochondria and synaptic vesicles. In contrast, treatment with a novel class of GSMs had no significant effect on these measures. Since knockdown of APP by specific siRNA prevented GSI-induced changes in BDNF axonal trafficking and signaling, we concluded that GSI effects on APP processing were responsible, at least in part, for BDNF trafficking and signaling deficits. Our findings argue that with respect to anti-amyloid treatments, even an APP-specific GSI may have deleterious effects and GSMs may serve as a better alternative.

## Introduction

Alzheimer’s disease (AD), characterized with β-amyloid peptide-containing neuritic plaques and Tau-containing tangles[1–6], is a neurodegenerative disorder leading to progressive cognitive decline and dementia with increasing impairment of daily functions[3, 7–12]. To date, there are no disease-modifying treatments for this fatal illness.

**Fig 1:**
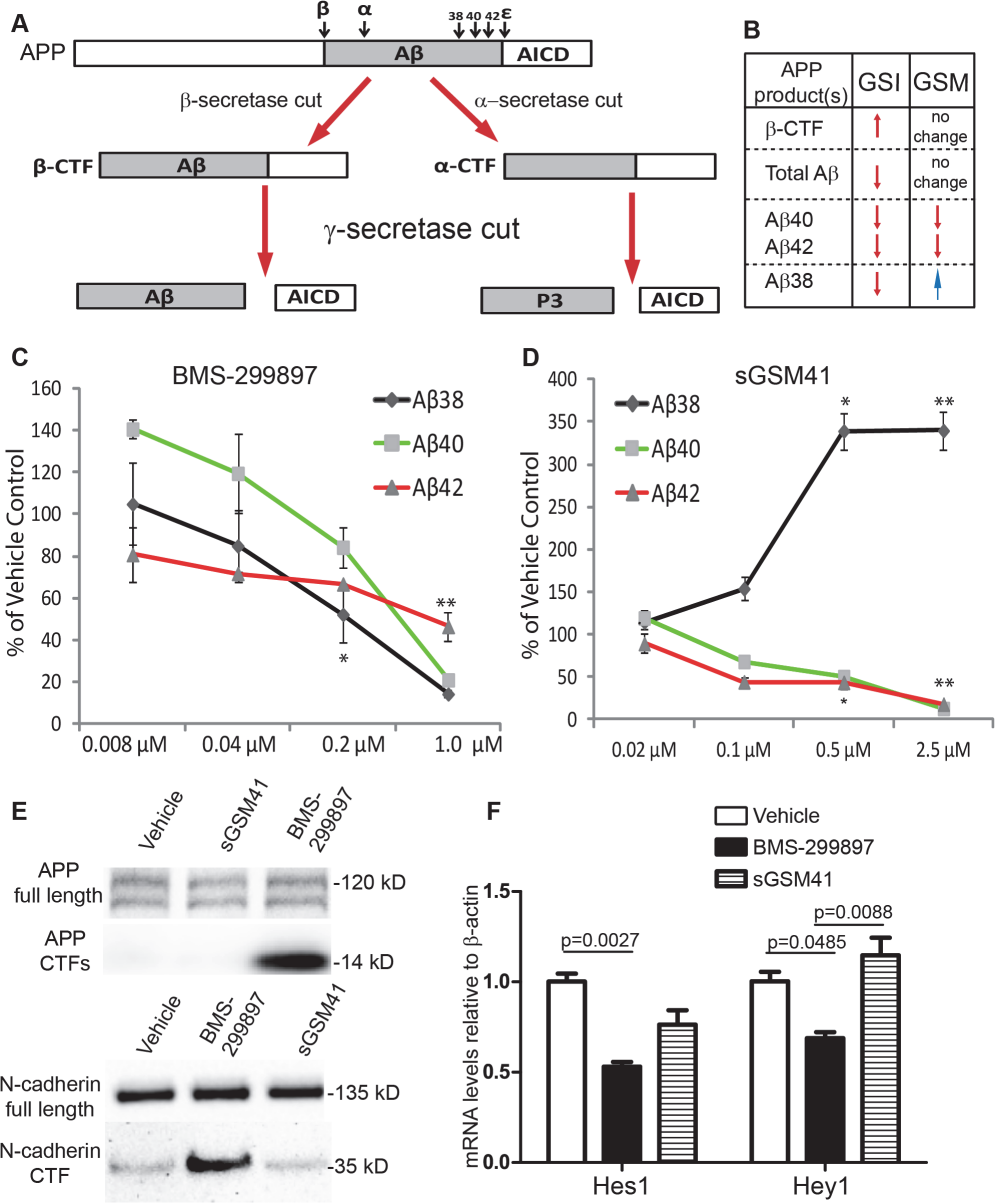
Differential effects of BMS-299897 and sGSM41 on APP processing. **A**: A diagram depicts APP processing and the pathways that GSI or GSM treatment differentially affects Aβ peptide formation and the production of APP C-terminal fragments (APP CTFs). First, β-secretase or α-secretase cleaves APP, leading to the production of either β-CTF or α-CTF. Cleavage of β-CTF by γ-secretase at multiple sites yields several Aβ peptides and the APP intracellular domain (AICD). Cleavage of α-CTF by γ-secretase gives rise to and AICD and the P3 fragment. **B**: Differential effects of GSI and GSM on the production of Aβ species and APP β-CTF [34–36]. Rat E18 cortical neurons (DIV7) were treated with GSI BMS-299897 (**C**) or sGSM41 (**D**) for 24 hrs. The media were harvested and levels of Aβ species (Aβ38, 40, 42) in the media were measured as described in Materials and Methods (n = 3, *P < 0.05, **P < 0.01 using student’s *t*-test). Treatment with 1μM BMS-299897 or 2.5μM sGSM41 showed the most robust effect and these conditions were used in all other experiments herein. **E**: Western blotting analyses showing the processing of two substrates of γ-secretase, APP and N-cadherin, in cortical neurons treated with vehicle (0.1% DMSO), 1μM BMS-299897, 2.5μM sGSM41 for 24 hrs. **F**: Quantitative measurement of mRNA levels by real-time PCR in cortical neurons treated as in **E**. The mRNA levels are normalized to mRNA levels of β-actin (n = 3, mean±S.E., p values represent results of Student’s *t*-test).

Attempts to develop treatments have been informed by neuropathological, genetic, animal modeling and cell biological observations [9–11, 13–22]. All these sources point to amyloid precursor protein (APP) and its processing as significant for pathogenesis and to APP processing as a potential target for treatments[3, 12, 21, 23]. One potential target(s) is the processing of APP that leads to the production of amyloid β peptides (Aβ peptides), which requires the sequential cleavage of APP by β-secretase and γ-secretase[9–12, 18, 21]. The 40 and 42 residue-long Aβ peptides, Aβ_40_ and Aβ_42_, are the principal components of amyloid plaques (**Fig. 1A**). A large body of cell biological and animal model data has suggested that an increased Aβ42 to 40 ratio may modulate the structure of toxic species and that excessive Aβ_40/42_ peptides induce AD-relevant changes in neuronal structure and function [1–6]. The molecular structure(s) that mediate neuronal effects and their mechanism(s) of action are under active investigation [9, 10, 13–18, 20, 24]. Soluble Aβ_40/42_ peptides, possibly as oligomers or in higher order assemblies, may contribute to Aβ toxicity [3, 9–11, 14, 24–33].

Current efforts in drug development have targeted eliminating/reducing the production of Aβ_40_ and Aβ_42_ [2, 7, 8, 32–34, 37, 38]. One approach involves the use of γ-secretase inhibitors (GSIs) to prevent production of all Aβ peptides [39–43]. However, Phase III clinical trials using the GSI, semagacestat, were discontinued due to detrimental impacts on both cognition and daily function[7, 8, 35]. Although the mechanism for the deleterious effect was not defined, the emergence of clinical findings suggests that inhibition of Notch processing by GSI contributed to these effects, arguing for the development of Notch sparing GSIs [43–45]. Of note, however, worsening of cognition by both semagacestat and a reportedly Notch-sparing GSI was recently demonstrated in the AD mouse model of Tg2576 as well as in wild type mice [46]. An alternative approach for reducing Aβ_42_ and Aβ_40_ levels is to enhance, rather than inhibit, the activity/processivity of γ-secretase via modulators of this enzyme complex, a class of small molecules termed γ-secretase modulators (GSMs) (**Fig. 1B**)[34, 36, 43]. GSMs have been shown to decrease levels of Aβ_42_ and Aβ_40_ while increasing the levels of shorter Aβ peptides, such as Aβ_38,_ without affecting total Aβ levels [34, 46–50]. Because shorter Aβ peptides are viewed as non- or less pathogenic [9–11, 20, 35, 36, 43], GSMs are being pursued as potential anti-amyloid therapies.

As yet the data for GSI and GSM effects on neurons is limited, leaving uncertain what impact they might have on the structure and function of neurons. To explore this topic we examined GSI and GSM treatments on a number of neuronal phenotypes. Among which, neuronal trafficking and signaling of brain-derived neurotrophic factor (BDNF) plays a critical role for the development and maintenance of neuronal circuits[51–58]. Herein, using cultures of cortical and hippocampal neurons, we examined axonal trafficking and signaling of BDNF and other selected phenotypes. As the GSI, we used the arylsulfonamide-based BMS-299897[59], a member of GSI family that includes the Notch sparing GSI avagacestat [7, 41]. The GSM, sGSM41[36], a methylimidazole-containing arylaminothiazole, is one of a series of closely related GSM chemotypes [60] that shows increased solubility, ergo the designation sGSM [36]. Our studies provide evidence that GSIs, but not GSMs, disrupted BDNF axonal trafficking and signaling and induced changes in neuronal morphology.

## Materials and Methods

### Ethics Statement

All surgical and animal procedures are carried out strictly according to the NIH Guide for the Care and Use of Laboratory Animals. All experiments have been approved by the University of California San Diego Institutional Animal Care and Use Committee (Protocol# S09371).

### Antibodies and Reagents

Rabbit antibodies against ERK1/2, pAkt1 were from Cell Signaling Technologies. Rabbit polyclonal IgGs against phospho-TrkB (pY490) were kindly provided by Dr Moses Chao (NYU), the mouse monoclonal IgGs against TrkB were from BD. Mouse monoclonal IgGs against pErk1/2 (E4) were from Santa Cruz. Other antibodies used include: rabbit polyclonal IgGs against pCREB(ser133) (Cell Signaling), mouse monoclonal IgGs against β-actin, β-tubulin, MAP2 (Sigma). C-terminal APP rabbit monoclonal IgGs (Epitomics), rabbit polyclonal IgGs against the C-terminal fragment of APP[34], mouse IgGs against N-cadherin (clone C36) (BD). Rabbit monoclonal IgGs against Akt1 were from Abgent. Rabbit anti-β-actin antibodies were from Rockland.

Mouse IgGs against Tau were from Millipore. Mouse IgGs against GAPDH were from GenTex. Secondary antibodies conjugated to Alexa for immunostaining, quantum dots 655-streptavidin conjugates (QDs) for live imaging of axonal transport of BDNF were from Invitrogen. Unlabeled human recombinant BDNF was from Genentech. All culture media, antibiotics and buffers were from Life Technologies.

The GSI BMS-299897 and the GSM sGSM41 were supplied by Dr S. L. Wagner of UCSD. BMS-299897 is an arylsulfonamide, whose core structure is shared with the Notch sparing GSI avagacestat[34, 36, 46]. The sGSM41 (International patent WO 2011/163636 A2)[36], a methylimidazole-containing arylaminothiazole, is one of a series of closely related GSM chemotypes[34]. Both BMS-299897 and sGSM41 were prepared in dimethyl sulfoxide (DMSO). 0.1% DMSO (final concentration) was used as the vehicle control for all experiments.

### Primary neuronal cultures and microfluidic chamber cultures

Rat E17–18 primary embryonic mixed cortical and hippocampal neurons were dissected and maintained as described [57]. Two-thirds of the media was replaced every 2–3 days until experiments. For imaging experiments, hippocampal neurons were cultured into microfluidic chambers with 450μm microgrooves (Xona microfluidics)[57]. For biochemistry experiments, cortical neurons were cultured in home-made microfluidic biochemistry chambers with 450μm microgrooves. For signaling studies, mass cultures of cortical neurons were starved for 2hrs in neurobasal media and were stimulated with 50ng/ml BDNF. Neurons were harvested and lysed in PBS containing 1% NP-40, 0.1% SDS, 0.1% deoxylcholate, 1mM PMSF (phenylmethylsulfonyl fluoride), 0.2mM sodium orthovanadate and protease inhibitor cocktail (Roche). Axonal signaling assays were performed similarly except that BDNF was added only to the distal axon chambers. Axonal lysates were collected by adding a small volume of lysis buffer (15–20 μl) into the distal compartments.

### Drug treatments and siRNA transfections

Neuronal cultures were treated at indicated DIVs for 24 hrs with 1μM BMS-299897, 2.5μM sGSM41 or the vehicle DMSO (final concentration: 0.1%) for most of the experiments, or for different periods of time as indicated in specific experiments. Transfections of siRNAs were performed on ∼100,000 neurons at DIV4 using NTER Nanoparticle transfection system (Sigma) following the protocol provided. The siRNAs used were: 1) siRNA against rat APP (Sigma: Product# PDSIRNA2D: SASI_Rn01_00086595 with starting sequence at 366), and 2) the MISSION siRNA Universal Negative Control #1 (Sigma: Cat#SIC001). Knockdown experiments were optimized by examining both mRNA and protein expression of APP. A knockdown efficiency of ∼80% at the mRNA level as quantitated with the 7300 Real Time PCR System (Appiled Biosystems) and ∼30% at the protein level by immunoblotting was routinely achieved.

### Live Imaging of Quantum dots-labeled BDNF (QD-BDNF)

**Fig 2:**
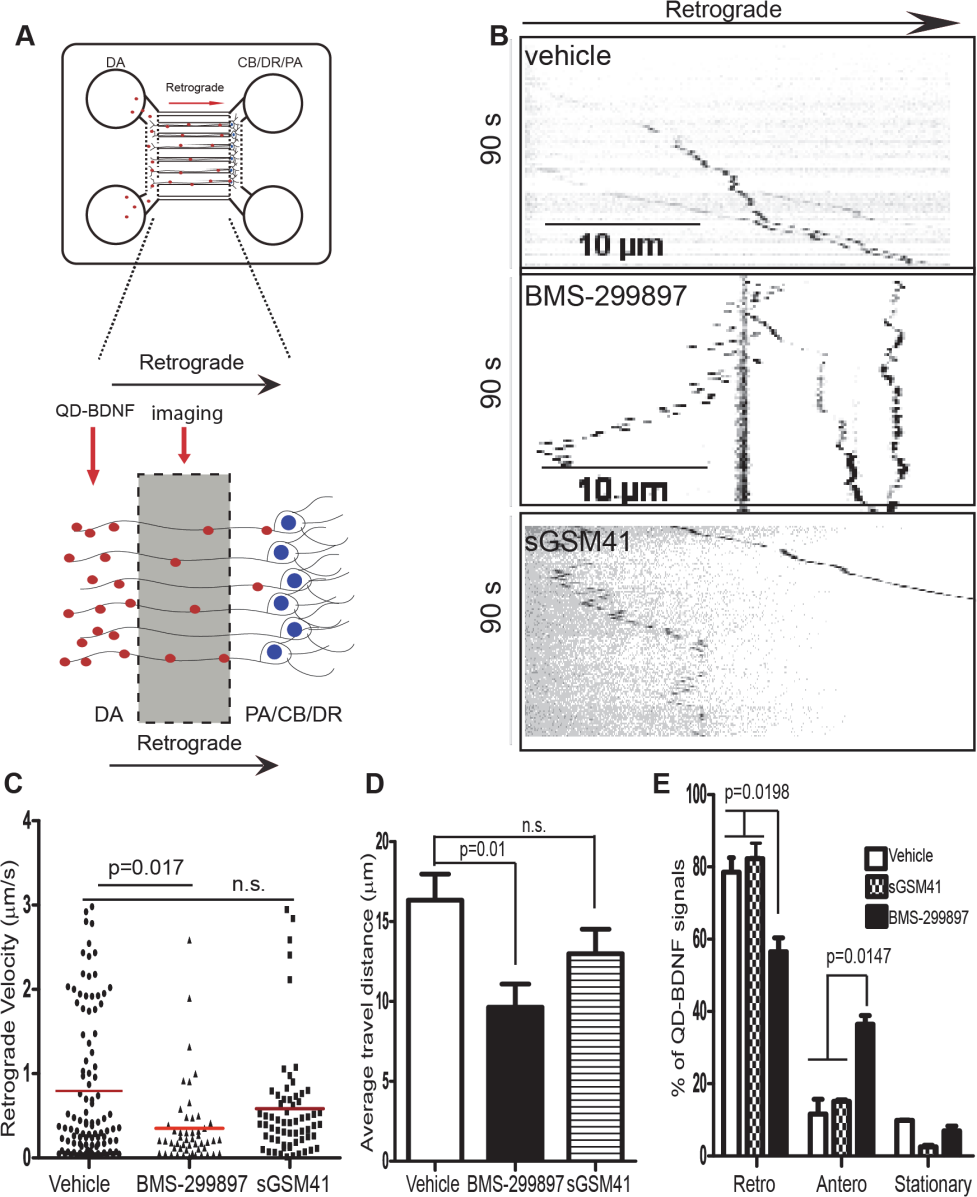
BMS-299897, not sGSM41, induces deficits in retrograde axonal trafficking of QD-BDNF. **A**: Microfluidic chamber cultures of rat E18 hippocampal neurons for live imaging of axonal transport of QD-BDNF. At DIV7, neurons were treated with vehicle, BMS-299-897 or sGSM41 followed by live imaging as described in Materials and Methods. DA: distal axon; CB: cell body; DR: dendrites; PA: proximal axon. **B**: Representative kymographs generated from a 90 sec time lapse series (captured at 1frame/s) of axonal movement of QD-BDNF are shown. **C**: Distribution of instantaneous velocities of individual QD-BDNF molecules moving in the retrograde direction under each treatment condition with the mean values marked as red horizontal lines. Time-lapse recordings were collected from at least three independent experiments for each treatment conditions with 15–20 separate movies being collected blindly from each sample. The data represent 70–125 QD-BDNF molecules. **D**: Analysis of the average distance travelled by QD-BDNF molecules for each treatment condition. **E**: Breakdown of transport directionalities (Retro: retrograde, Antero: anterograde, and Stationary) of all QD-BDNF signals under each treatment condition. The percentiles for each direction are shown. The data in **D**, **E** represent mean±S.E from three independent experiments. All p values were calculated using student’s *t*-test.

Monobiotinylated BDNF was prepared in a method similar to that described previously [61]. QD-BDNF, which signals and transports like normal BDNF was prepared as described [57]. We chose QD655 with an emission spectrum that can be visualized in the TexasRed channel. Following starvation for 2 hrs in neurobasal media, QD-BDNF (0.1nM) was added to the distal chamber and allowed to incubate for 2 hrs as depicted in **Fig. 2A**. All images were captured on a Leica DMI6000B inverted microscope with a 100x oil objective lens with a CCD camera (Rolera-MGi Fast 1397 from Qimaging). The microscope is equipped with an environmental chamber (37°C, 5% CO_2_). Images were collected at 1 frame/s for a total of 2 min per movie. Ten to twenty movies were collected/condition. For each condition, 75–130 QD-BDNF signals from at least two independent experiments were analyzed. Kymographs generated from each movie were analyzed using the MetaMorph Software. Direction of movement was determined by the angle measurement for each line on kymographs using cut-offs as specified below: retrograde (-1 to -89.4), anterograde (<-90.6), and stationary (90±0.5). All live-imaging experiments were performed blindly to minimize bias.

### SDS-PAGE, Western blotting

Equal amounts of protein samples were run on 4–12% Novex precast gels (Life Technologies). For APP CTF detection, 4–20% precast gels were used. Immunoblotting was performed following standard practice [62]. All blots were developed using the BioRad Clarity Western ECL substrate and visualized on a BioRad ChemiDoc XRS+ with the BioRad ImageLab software. All quantification was taken from blots within the linear range of exposure and all analyses were performed using the ImageLab software (BioRad).

### Aβ Meso scale assay

Aβ38, 40, and 42 were detected as previously described [34]. Briefly, 500K embryonic cortical neurons were treated in triplicate in 400μl of media containing various concentrations of drugs or vehicle. Media was collected 24 hrs later and Aβ peptides in the media were detected using the Aβ triplex Meso scale assay (Meso Scale Discovery). The amount of Aβ peptides was presented as percentage of vehicle control.

### mRNA extraction and quantification

mRNA levels of Hes1 and Hey1 were measured using cDNA synthesized from total RNAs (RNAeasy Micro Kit from Qiagen). Quantitative real-time PCR reactions in triplicate were performed for each sample using transcript-specific primers (Qiagen). Minus reverse transcriptase (-RT), minus template, and minus primer controls were included to minimize background signals. Melting curve analyses were performed to ensure the specificity and quality of the qPCR amplifications. Expression of Hes1 and Hey1 was calculated using the comparative threshold (ΔΔCT) method and normalized to GAPDH.

### Immunostaining, mitochondria and synaptic vesicle analysis, and electron microscopy

For mass cultures, neurons were maintained on Poly-L-Lysine-coated coverslips and were fixed in 4% paraformaldehyde (PFA) in PBS for 15 min. Neurons were then permeabilized in 0.2% Triton X-100 in PBS. Samples were blocked using 3% BSA, 5% goat serum in PBS for 1 hr at room temperature. Primary antibodies were added in block solution for 1 hr at room temperature or overnight at 4°C. Secondary antibodies were added in 3% BSA for 1 hr at room temperature. Coverslips were washed and mounted for analysis by microscopy. Images were captured using Leica DMI6000B inverted microscope with a 100x oil objective lens and were processed using ImageJ.

Treated neurons were fixed and stained for pCREB with a specific antibody (Cell Signaling). Images were taken with a 20x objective lens by scanning the cells directly adjacent to the microgrooves. ImageJ was used to set the threshold for all pCREB images equally. To determine the percentage of nuclei that were positive for pCREB, the “analyze particles” function in ImageJ was used to automatically count the number of Hoechst-stained nuclei and the number of pCREB-positive nuclei.

Deep Red FM Mitotracker (Molecular Probes) was used to visualize mitochondria following the manufacture’s protocol. A rabbit polyclonal synaptophysin antibody (GenTex) was used to stain fixed neurons on coverslips. All images were acquired and analyzed blindly. For EM analysis, neurons were fixed and sectioned at the Cellular and Molecular Medicine Electron Microscopy Facility at UCSD following a standard EM flat embedding protocol. Images were acquired on a FEI Tecnai Spirit G2 BioTWIN Transmission Electron Microscope and were processed using ImageJ.

### Statistical analysis

All experiments were repeated at least three times independently. Statistical analyses of results and calculation of *p* values were performed using Prism5 (GraphPad Software, La Jolla, CA). For pairwise comparisons, the Student’s *t*-test and Mann-Whitney tests were used. For multiple comparisons, the Tukey one way ANOVA test was used.

## Results

### BMS-299897 and sGSM41 induced markedly different effects on processing of APP and other γ-secretase substrates

GSIs inhibit production of all Aβ species while increasing the β-carboxyl terminal fragment of APP i.e. β-CTF [34–36, 46] (**Fig. 1B**). Unlike GSIs, GSMs selectively prevent the production of Aβ_42_ and Aβ_40_ with a concomitant enhancement of Aβ_38_ production [34–36]. Furthermore, GSMs do not appear to cause accumulation of β-CTF [34–36, 46] (**Fig. 1B**). To confirm the differential effect of BMS-299897 and sGSM41 treatment on APP processing in neurons, we measured the levels of Aβ_42,_ Aβ _40_ and Aβ _38_, as described in Methods and Materials. BMS-299897 reduced the levels of each of the Aβ peptides. At ∼1.0 μM, BMS-299897 decreased these peptides to levels ranging from 20 to 50% of the vehicle control (**Fig. 1C**). sGSM41, on the other hand, preferentially reduced Aβ_42_ and Aβ_40_ while increasing the levels of Aβ_38_ (**Fig. 1D**). Consistent with previous results [34, 36], the total level of Aβ peptides was not markedly changed by sGSM41, thus there was no apparent inhibition of γ-secretase by sGSM41. At 2.5μM, sGSM41 essentially eliminated all Aβ_42/40_ species while increasing Aβ_38_ to ∼300% of the level of vehicle controls. Based on these results, we elected to use a final concentration of 1.0 μM for BMS-299897 and 2.5μM for sGSM41 in all remaining studies.

To further examine the differences in APP processing, we assessed the impact of BMS-299897 and sGSM41 on the levels of APP CTFs. In agreement with published reports [34, 59], treatment of cortical neurons with BMS-299897 increased APP CTFs while sGSM41 treatment did not (**Fig. 1E**). These results show that BMS-299897 and sGSM41 exert different effects on processing of endogenous APP in neuronal cultures.

In addition to APP, γ-secretase acts on a large number of substrates [43, 50, 63, 64], among which are N-cadherin and Notch[39, 65–68]. We asked if the inhibition of APP processing, seen with the BMS-299897 but not with the sGSM41, would be seen with these two substrates. N-cadherin processing was dramatically inhibited by the BMS-299897, but not the sGSM41 (**Fig. 1E**). Inhibition of γ-secretase has been shown to increase the level of CTFs of Notch and to impede the translocation of the Notch intracellular domain (NICD) to the nucleus, thus inhibiting downstream transcriptional events such as the expression of transcription factors, Hes1 and Hey1 [46, 66, 68]. We tested BMS-299897 and sGSM41 effects on the expression of these two factors by quantifying their mRNA levels. Treatment with BMS-299897, but not sGSM41, significantly decreased Hey1 and Hes1 mRNA levels in cortical neurons (**Fig. 1F**). These data are evidence that BMS-299897 is not Notch sparing at the concentration used in our studies. Taken together, the results show that BMS-299897 and sGSM41 are markedly different in their effects on the processing of γ-secretase substrates in neurons. They confirm inhibition of APP processing by BMS-299897 and enhanced processing of APP by the sGSM41.

### BMS-299897, but not sGSM41, induced deficits in retrograde axonal transport of QD-BDNF in hippocampal neurons

Increasing evidence suggests that accumulation of β-CTF (the carboxyl terminal 99 residues of APP, also known as C99) disrupts the endocytic pathway [69] and negatively impacts neuronal function [42, 70–79]. Given the different effects detected for BMS-299897 and sGSM41 on processing of APP, and the marked increase in β-CTFs induced by BMS-299897 but not by sGSM41 (**Fig. 1E**), we tested for possible effects on axonal trafficking of BDNF-containing endosomes. We measured retrograde axonal transport of quantum-dot conjugated BDNF (QD-BDNF) in hippocampal neurons grown in compartmentalized microfluidic chambers, as described previously[55–57] (also see **Fig. 2A**). Axonal movement of QD-BDNF in neurons treated with vehicle, BMS-299897, sGSM41 was captured by live cell imaging (**S1–S3 Movies**). Analysis of the corresponding kymographs (**Fig. 2B**) revealed significant differences. Vehicle treated cultures demonstrated persistent, processive movement of QD-BDNF, a pattern fully consistent with earlier findings [55, 57]. In contrast, the BMS-299897-treated cultures showed, in addition to processive movement, a much greater number of QD puncta that moved anterogradely, or switched between anterograde and retrograde trajectories (**Fig. 2B**). The pattern of QD-BDNF movement in sGSM41-treated cultures much more closely resembled the vehicle, with relatively few stationary or anterogradely moving puncta. **Fig. 2C** plots the instantaneous retrograde velocity – i.e. the velocity while moving; the average for vehicle controls was ∼0.8 μm/s, a value consistent with earlier observations[55, 57]. The distribution of instantaneous velocities was lower in BMS-299897-treated neurons with the average speed of ∼0.3 μm/s, a difference that was significant (p = 0.017). The distribution of instantaneous velocities in the sGSM41-treated cultures was similar to the vehicle-treated neurons and the average was not significantly different. We further calculated the average distance travelled by QD-BDNF. This value was significantly reduced by about 40% for BMS-299897-treated samples as compared to the vehicle control (**Fig. 2D**). Though there was a trend to lower values in sGSM41-treated neurons, the difference was not statistically significantly (**Fig. 2D**). To define the directionality of axonal movement of QD-BDNF, we sorted and quantified the percentile of individual QD-BDNF molecules moving retrogradely, anterogradely, or remaining stationary as defined in the Materials and Methods. We observed that in comparison to the vehicle control, BMS-299897 treatment reduced the portion of QD-BDNF signals moving in the retrograde direction (p = 0.0198) with a concomitant increase in the portion of signals moving in the anterograde direction (p = 0.0147) (**Fig. 2E**). In contrast, directionality was not significantly affected by sGSM41 treatment (**Fig. 2E**). These findings show that the GSI, but not the sGSM, disrupted axonal transport of BDNF.

### BMS-299897, but not sGSM41, inhibited TrkB-mediated signaling in neuronal cultures

**Fig 3:**
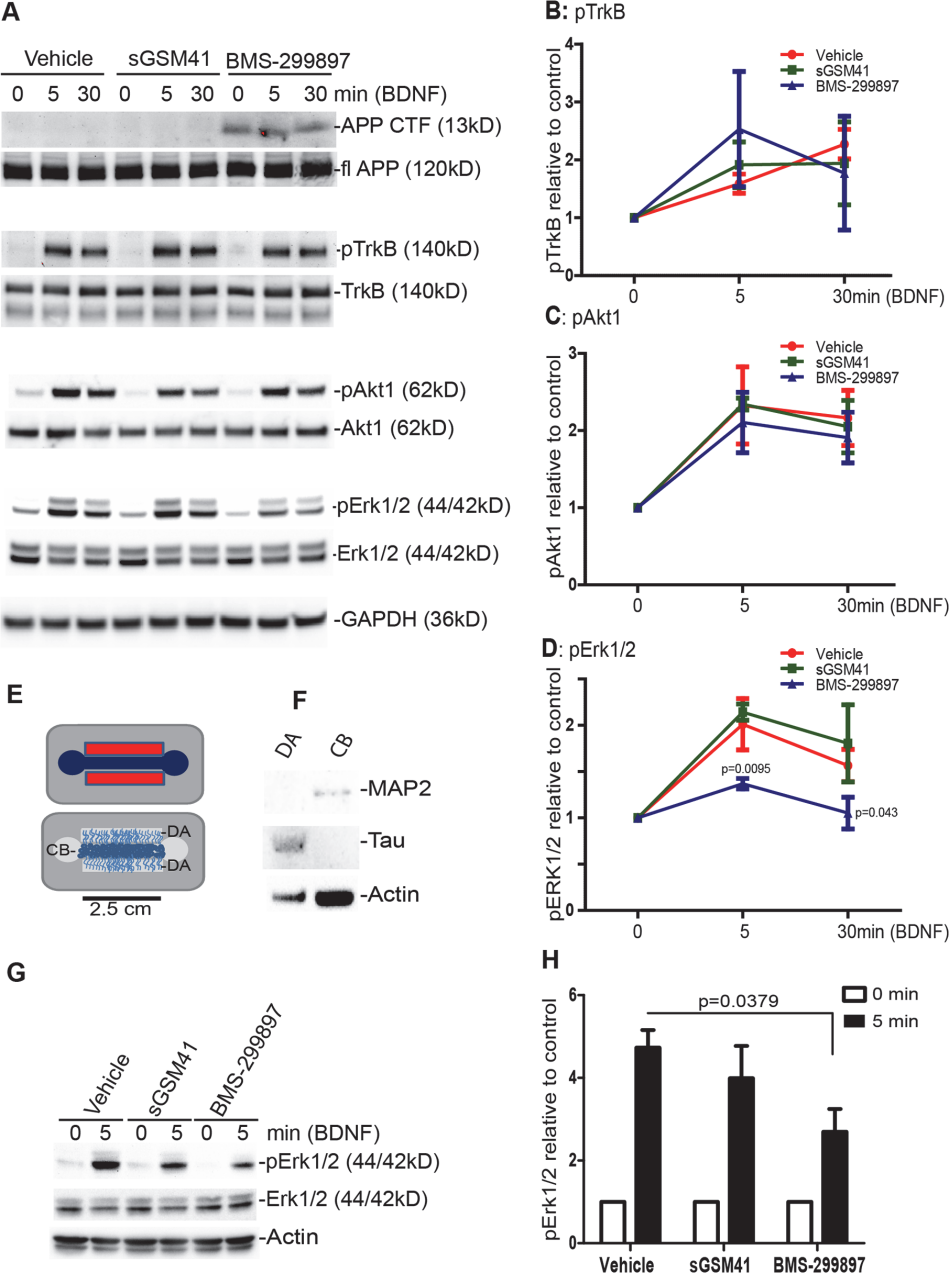
Differential effects of BMS-299897 and sGSM41 on BDNF/TrkB-mediated signaling pathways. Rat E18 cortical neurons were either cultured in 12-well plate (mass cultures) (**A-D**) or in microfluidic biochemistry chambers (**E-H**) to treat and harvest axons only. Neurons were pretreated with vehicle, BMS-299897, sGSM41 for 24 hrs followed by stimulation with 50ng/ml BDNF as indicated. Equal amounts of protein lysates were analyzed by SDS/PAGE and immunoblotting with specific antibodies as indicated (**A**). Semi-quantitative results are shown for the level of activated TrkB (pTrkB in **B**), activated Akt1 (pAkt1 in **C**) and activated Erk1/2 (pErk1/2 in **D**) (mean±S.E., n = 3 for pTrkB, n = 5 for pAkt1, n = 5 for pERK1/2, *P = 0.04, ***P<0.001 using Student’s *t*-test). **E**: Diagram of microfluidic biochemistry chambers used for axonal signaling assays. **F**: Western blotting analyses of lysates from the distal axon chamber (DA) and cell body chamber (CB). Samples from CB samples are enriched for the dendritic protein, MAP2 and samples from DA are enriched for Tau. Total samples from the distal chambers were compared against 1/4^th^ of lysates from the cell body chamber collected from the same experiment. Both CB and DA chambers were pretreated with vehicle, sGSM41 or BMS-299897 and only distal axonal chambers were treated with BDNF (50ng/ml) for 0, 5 min. **G**: Axonal lysates were analyzed by SDS-PAGE/immunoblotting for the level of pErk1/2, total Erk1/2 and β-actin. The results in G that are normalized against β-actin (mean±S.E., n = 3, *P<0.05) are shown in **H**. All signaling data analyzed are from 3 or more separate experiments.

Given that BMS-299897 induced deficits in BDNF axonal transport, this raised the possibility that the compound might also impact BDNF signaling. We thus examined BDNF signaling in both mass cultures and compartmentalized cultures of E18 rat cortical neurons; the choice of cortical neurons was based on the ability to harvest sufficient amounts of protein lysates. As expected and consistent with earlier findings[34, 46], treatment with BMS-299897, but not sGSM41, induced accumulation of APP CTFs, while having no apparent impact on the level of full length APP in mass cultures (**Fig. 3A**). We then examined by immunoblotting activation of the TrkB receptor and two downstream signaling pathways, Akt1 (part of the Akt/PI3K signaling cascade) and Erk1/2 (part of the MAPKK signaling cascade). Following addition of BDNF (50ng/ml), early and sustained activation of TrkB was observed after both BMS-299897 and sGSM41 treatments. The responses varied somewhat across cultures, but there was no significant difference in pTrkB levels in comparison with the vehicle control (**Fig. 3A and B**). Activation of Akt1 was also equally robust under all treatment conditions (**Fig. 3A and C**). However, activation of Erk1/2 did distinguish the treatments in that BMS-299897-treated neurons responded less robustly than the vehicle at both 5 and 30 minutes (**Fig. 3A and D**). The response to BDNF signaling with the sGSM41-treated samples was not significantly different from the control (**Fig. 3A and D**).

To explore further the possibility that disrupted trafficking of BDNF in axons was correlated with changes in signaling, we used a 3-chamber microfluidic device in which adequate amounts of axonal protein could be extracted (**Fig. 3E**). Axons were treated with 50ng/ml BDNF for 30 min followed by immunoblotting lysates from distal axons (DA). As indicated, lysates from this compartment were enriched for Tau, an axonal marker; correspondingly, these lysates were essentially devoid of MAP2, a dendritic marker protein that was present in lysates of the cell body chamber (CB) which also contain dendrites (**Fig. 3F**). BMS-299897 treatment elicited a much less robust of activation of ERK by BDNF at 5 min, as compared to responses in cultures treated with either vehicle or sGSM41 (**Fig. 3G and H**). These findings are evidence that the GSI, but not the GSM, negatively impacted BDNF-induced Erk1/2 signaling.

### BMS-299897, but not sGSM41, induced changes in organelles present in neuronal processes

**Fig 4:**
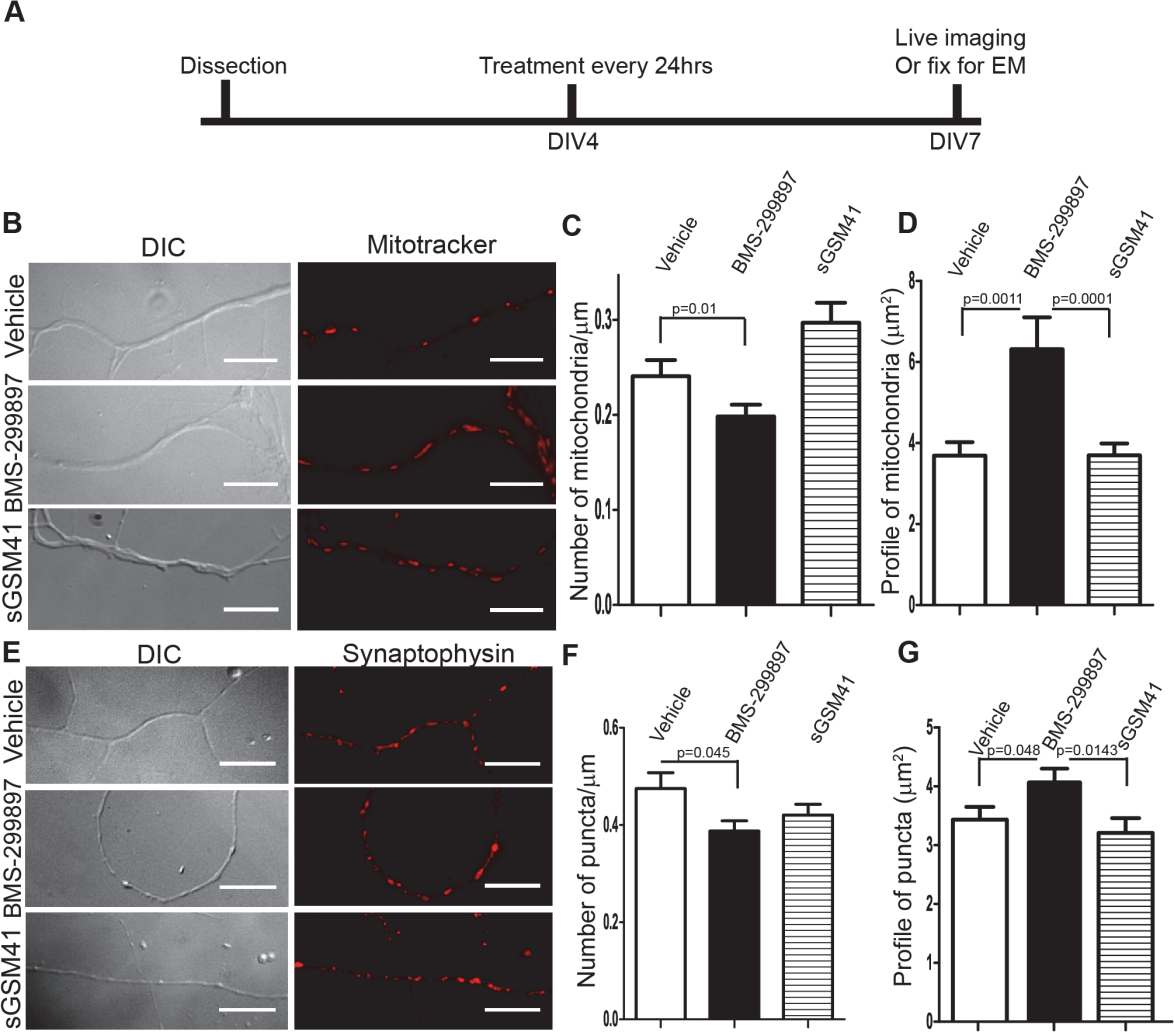
BMS-299897, not sGSM41, alters the distribution of mitochondria and synaptophysin-positive vesicles within processes of rat E18 hippocampal neurons. **A**: Experimental design for examining distribution of mitochondria and synaptic vesicle precursors within neuronal processes. Rat E18 hippocampal neurons at DIV4 were treated every 24 hrs with vehicle, BMS-299897 or sGSM41. Neurons at DIV7 were either labeled with MitoTracker for analysis of mitochondria (**B-D**) or fixed and stained for synaptophysin with a specific antibody (**E-G**). **B**: Representative images of DIC and MitoTracker under each treatment conditions. The density of mitochondria (**C**) and the measurement of mitochondrial profile (**D**) are quantitated and presented (n = 15–20 neurites for vehicle, sGSM41 samples. For BMS-299897-treated samples, n = 95–150 puncta, mean±S.E., ***P<0.001). **E**: Representative images of DIC and synaptophysin immmunostaining for each treatment condition. The density of mitochondria (**F**) and the measurement of mitochondrial profile (**G**) are quantitated and presented (For density analysis, n = 29–40 neurites, mean±S.E., *P<0.05. For profile measurement, n = 26–27 puncta). Scale bar = 20μm.

Given the changes in BDNF trafficking and signaling, it was of interest to explore whether GSI-induced deficits might be accompanied by structural changes in neuronal processes. We examined mitochondria and synaptophysin-positive vesicles. Neurons were treated every 24 hrs with vehicle, BMS-299897 or sGSM41 from DIV4 to DIV7 (**Fig. 4A**). Mitochondria were labeled with MitoTracker (**Fig. 4B**); the density of these puncta and their size, as judged by their apparent length, were then evaluated (**Fig. 4C and D**). Relative to the vehicle control, BMS-299897 treatment reduced mitochondrial density while markedly increasing length (**Fig. 4C and D**). Treatment with sGSM41 did not reduce density; indeed, there was a significant, albeit small, increase. Unlike BMS-299897, sGSM41 had no effect relative to the vehicle on mitochondrial length (**Fig. 4C and D**). In parallel studies, synaptophysin-vesicles were examined (**Fig. 4E**). BMS-299897 treatment reduced the density of synaptophysin-positive puncta and increased their apparent size (**Fig. 4F and G**). Treatment with sGSM41 had no significant effect on either of these measures (**Fig. 4F and G)**.

**Fig 5:**
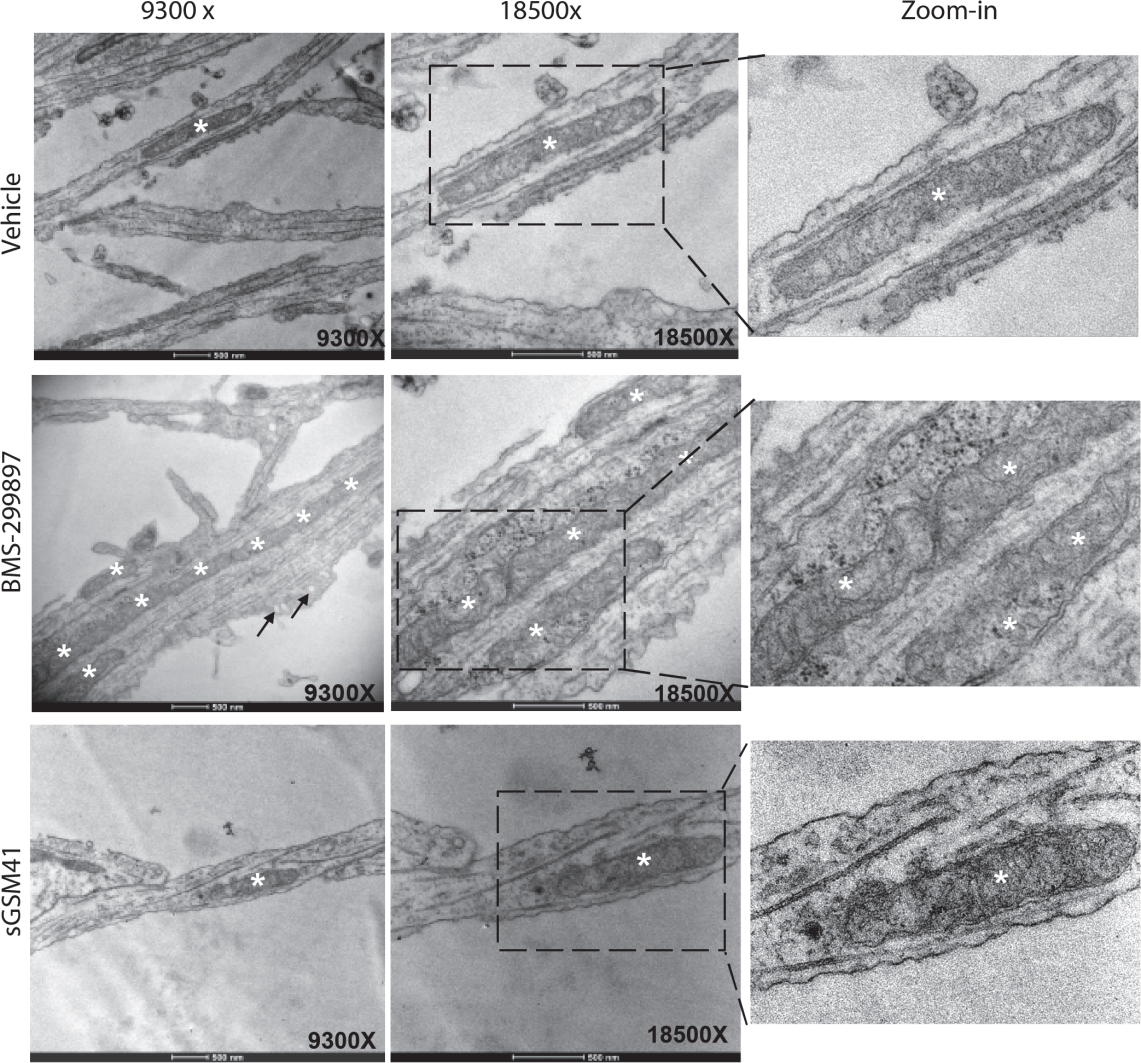
BMS-299897, not sGSM41, induces clustering of mitochondria within processes of rat E18 hippocampal neurons. Samples were prepared as in Fig. **4A**. The ultra-structures of hippocampal neurites were examined using transmission electron microscopy as described in Materials and Methods. For each treatment condition, representative images of 9300x, 18500x and Zoom-in are shown. Asterisk denotes a single mitochondrion, arrows point to vesicles. EM data were verified by two independent researchers at the Cellular and Molecular Medicine Electron Microscopy Facility at UCSD.

To further define the effects of BMS-299897 treatment, we examined neuronal processes *via* transmission electron microscopy (**Fig. 5**). Mitochondria, which were readily identified, were sparse in neurites of vehicle-treated neurons in low magnification (9,300x) images (**Fig. 5**). At high magnification (18,500x), mitochondria were localized along microtubules (**Fig. 5**). Neurons treated with sGSM41 showed no obvious differences in mitochondria as compared to vehicle at either 9,300x or 18,500x magnification (**Fig. 5**). In contrast, neurons treated with BSM-299897 displayed striking abnormalities. Specifically, there was abnormal accumulation of mitochondria (marked with * in **Fig. 5**). Zoom-in images of these stretches revealed that mitochondria were neither enlarged nor fused, but rather that separate mitochondria were crowded together (Zoom-in images in **Fig. 5**). These findings suggest that the apparent increase in size of mitochondria with fluorescent imaging was due to focal accumulation. Thus, GSI, but not GSM, induced changes in organelles that are present and actively trafficked in neuronal processes, suggesting that GSI effects extend to organelles in addition to BDNF-containing endosomes.

### Extended BMS-299897-, not sGSM41- treatment induced changes in the morphology of neurons

**Fig 6:**
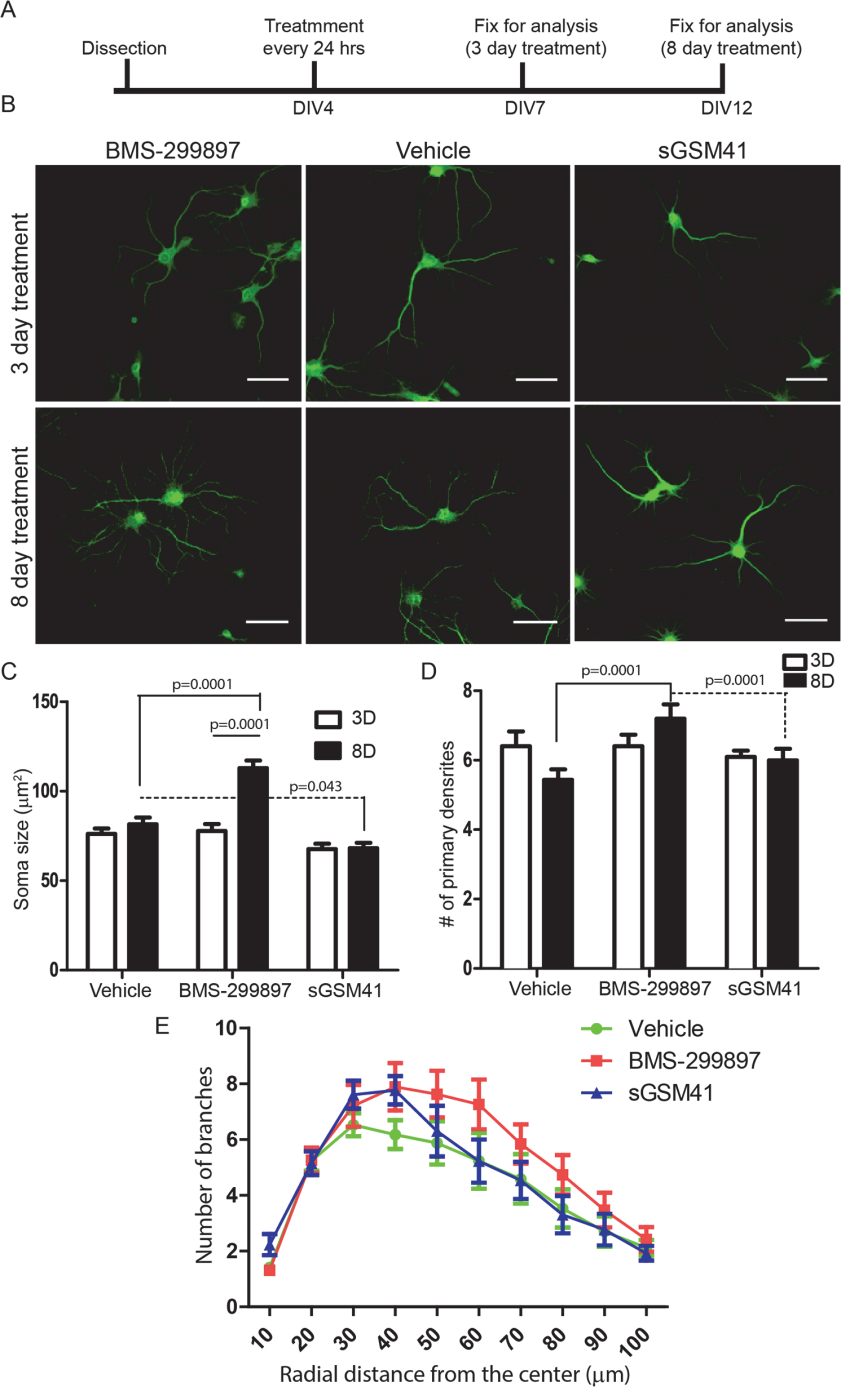
Sustained treatment with BMS-299897, but not GSM, causes marked changes in soma size and dendritic complexity in rat E18 hippocampal neurons. **A**: Experimental paradigm for examining the effect of sustained treatment with vehicle, BMS-299897 or sGSM41 on neuronal morphology of rat E18 hippocampal neurons. 3-day treatment and 8-day treatment were chosen to assess early and late changes in neuronal morphology. **B**: Representative images of MAP2 staining for each conditions at Day 3 or 8. **C**: Quantitative analysis of soma size (n = 25–30 neurons, mean±S.E., ***P<0.001). **D**: Quantitative analysis of number of primary dendrites (n = 17–25 neurons, mean±S.E, ***P<0.001). **E**: Sholl analysis of neuronal tracings for 8 day treatment with vehicle, BMS-299897 or sGSM41.

To examine the possibility that the GSI adversely affected other features of neuronal structure, we examined early and late stages of dendritic arborization in hippocampal neurons following treatment with vehicle, BMS-299897, or the sGSM41 for either 3 days (at DIV7) or 8 days (at DIV12) (**Fig. 6A**). Neurons were stained with MAP2 antibodies to visualize dendrites and neuronal somas (**Fig. 6B**). Treatment for 3 days resulted in no significant differences across treatment groups in gross morphology (**Fig. 6B**), soma size (**Fig. 6C**), or the number of primary dendrites (**Fig. 6D**). However, at 8 days of treatment, changes were evident in BMS-299897-treated neurons (**Fig. 6B**). Quantitative analysis revealed a marked increase in soma size for BMS-299897—treated neurons, as compared to either vehicle- or sGSM41-treated neurons (**Fig. 6C**). There was also an increase in the number of primary dendrites in BMS-299987-treated neurons (**Fig. 6D**). In addition, Sholl analysis showed a trend toward increased dendritic complexity in BMS-299897-treated neurons (**Fig. 6E**). Taken together, the data are further evidence for changes in neuronal morphology induced by GSI but not GSM.

### Reduced APP gene expression rescued axonal transport deficits induced by BMS-299897 treatment

**Fig 7:**
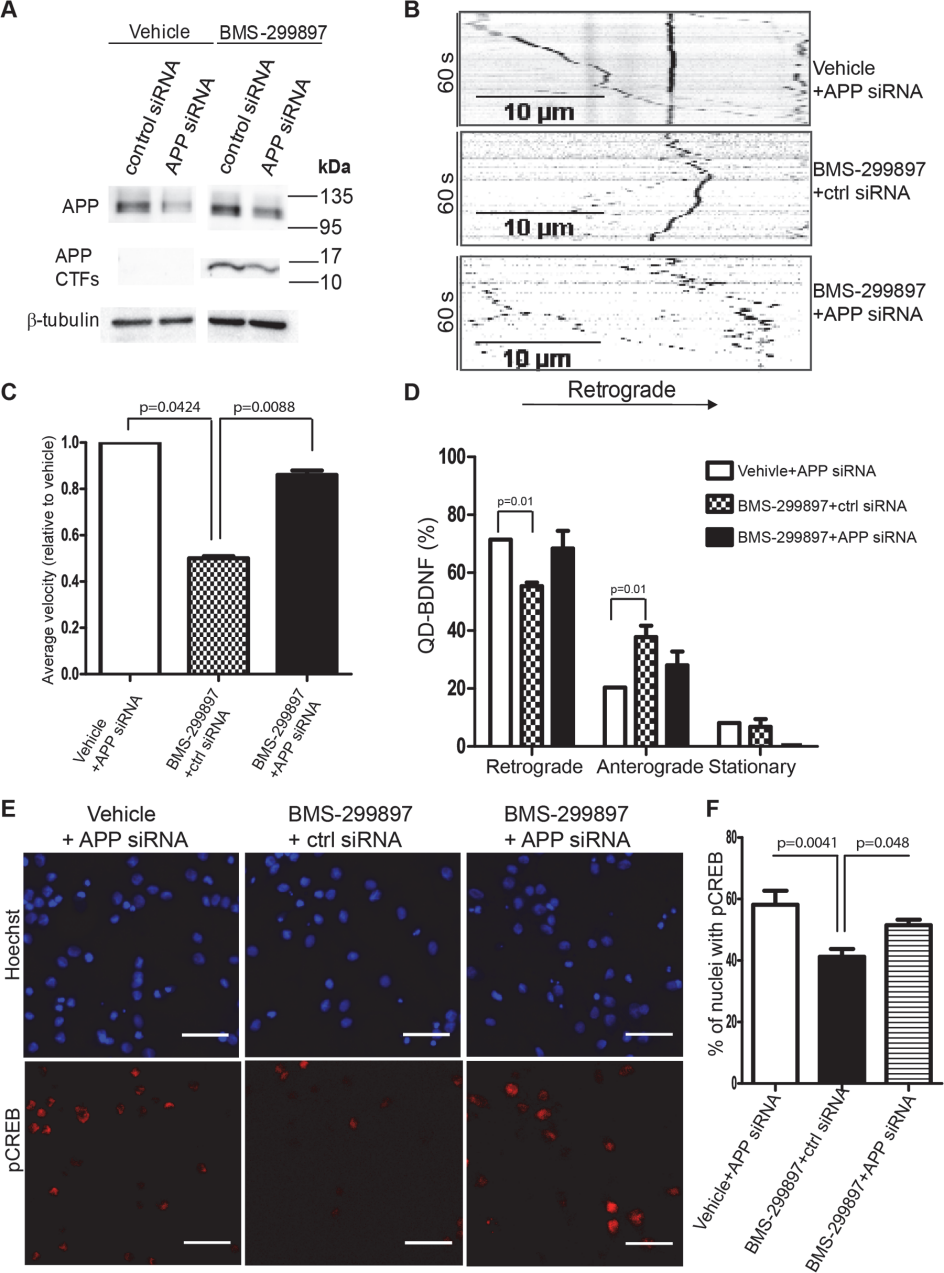
Knockdown of APP rescues deficits in velocity and directionality of axonally transported QD-BDNF induced by BMS-299897. **A**: Western blotting analysis of typical siRNA-mediated knockdown effect on full length APP and APP CTFs. Rat E18 hippocampal neurons (DIV5) were transfected with siRNA against APP or control siRNA for 72 hrs followed by drug treatment for 24 hrs. On average, the protein levels of APP and APP CTF were knocked down to 70% of normal levels. **B**: Live imaging of axonal transport of QD-BDNF was performed as in **Fig. 2**and representative kymographs of QD-BDNF movement (60 sec) within axons are shown for each condition. Retrograde direction is indicated. Analysis of QD-BDNF for average velocities (**C**) and directionality of QD-BDNF movement (**D**) revealed that knocking down APP prior to BMS-299897 treatment partially rescued the deficits in both the velocities and directionality of QD-BDNF as seen previously. 15–20 separate movies were collected and analyzed for each chamber. The data represent 50–130 QD-BDNF molecules (mean±S.E.). Neurons that were cultured in microfluidic chambers were transfected with siRNA and treated with BMS-299897 as in **A**. BDNF (50 ng/ml, final concentration) was added to the distal axon chambers only for 30 min. Neurons were fixed and stained for pCREB using a specific antibody. The nuclei were stained with the Hoechst dye. Representative images are shown in **E** and quantitative analysis of the percentage of nuclei that were pCREB-positive is shown in **F** (mean±S.E., n = 10 images, **P = 0.004). All images were obtained on a Leica DMI6000B inverted microscope (scale bar = 50μm).

In view of the fact that APP is a substrate for γ-secretase and that BMS-299897 and sGSM41 exhibited marked difference in APP processing(**Fig. 1E**) as well as earlier findings for the impact of the APP β-CTF on endocytic trafficking [1, 69, 80], we asked if BMS-299897 effect on APP contributed to axonal transport deficits. To address this possibility, APP protein levels were selectively reduced in hippocampal neurons using a specific siRNA prior to BMS-299897 treatment; axonal BDNF trafficking was then assessed. The siRNA for APP resulted in a decrease of ∼30% at the protein level as compared to the control siRNA (**Fig. 7A**). A similar reduction in APP CTFs was achieved when neurons were subsequently treated with BMS-299897 (**Fig. 7A**). In cultures treated with the vehicle together with either the siRNA for APP, or the control siRNA, there were no significant differences in the directionality of BDNF transport or the average retrograde velocity (**Fig. 7B-D; S4–S6 Movies**). In contrast, BMS-299897 treatment in the presence of the control siRNA resulted in marked changes in the directionality and velocity of movement relative to the vehicle controls; significantly, the differences were largely prevented in neurons treated with the siRNA for APP (**Fig. 7B-D; S4–S6 Movies**). Thus, in the presence of the siRNA for APP, BMS-299897 had no significant effect on either average velocity or the directionality of BDNF movement (**Fig. 7B-D; S4–S6 Movies**). Remarkably, the siRNA for APP also prevented the marked ∼50% decrease in the GSI effect on average velocity (**Fig. 7C**).

That reducing APP gene expression prevented the BMS-299897-mediated effects on BDNF axonal transport suggested that it would also prevent BMS-299897 effects on BDNF-mediated retrograde signaling. To explore this, neurons cultured in microfluidic chambers were treated with either APP siRNA or control siRNA followed by treatment with either vehicle or BMS-299897. BDNF was added only to the axonal chamber for 30 min; neuronal somas in the cell body chamber were then stained with a specific antibody to pCREB, a transcription factor that is translocated to the nucleus upon activation by axonal BDNF/TrkB signaling cascades[58, 81]. As predicted, neurons treated with BMS-299897 in combination with the control siRNA showed significantly less pCREB staining than neurons treated with vehicle + APP siRNA (**Fig. 7E and F**), a finding consistent with the earlier documentation of decreased axonal pErk1/2 levels (**Fig. 3G**). In contrast, the percentage of nuclei immunostained for pCREB in cultures treated with BSM-299897 plus the APP siRNA was not significantly different from those treated with the vehicle plus the APP siRNA (**Fig. 7E and F**). Thus, reducing APP expression, including the amount of APP CTFs, prevented changes in BDNF signaling induced by the GSI. We conclude that the effects of the GSI on axonal trafficking and signaling of BDNF was mediated, at least in part, by APP and/or APP CTFs.

## Discussion

The pathogenesis of AD is complex and likely multifactorial, with a diverse set of pathological signatures, including those for APP processing [3, 5, 9, 10, 12, 20, 27]. The many changes detected have brought different views as to how the disorder is caused and progresses. The early involvement of the endosomal pathway might contribute to pathogenesis due to either APP- or to tau-linked pathology [82–84]. Indeed, the endocytic pathway plays an important role in processing APP and in supporting transport of endocytic cargoes. Those cargoes carrying trophic signals from axons to cell bodies are known to play an important role in establishing, maintaining and modifying synaptic connections [85–88].

Our studies have established that GSI acts through altering APP processing to induce deficits of axonal trafficking and signaling of BDNF. They suggest an important role for APP in regulating endocytic trafficking and signaling of neurotrophic factors. Our findings are consistent with a recent study demonstrating that APP directly interacts with the two surface receptors for nerve growth factor (NGF): TrkA, p75^NTR^ to mediate endocytosis and signaling by NGF and its receptors[89]. Significantly, the study has demonstrated that downregulation of APP leads to reduced cell surface levels of TrkA/p75^NTR^ and results in inhibition of neurite outgrowth by NGF[89]. Therefore, it is possible that APP impacts neuronal function by regulating endocytic trafficking[80, 89].

Given the role for Aβ peptides, Aβ_42_ in particular, in both the pathology and pathogenesis of AD, recent attempts used GSIs to reduce Aβ_42_ levels. Many GSIs have shown the ability to markedly reduce the levels of Aβ_42_ as well as shorter Aβ peptides [38–40, 43, 44, 68, 90, 91]. However, patients treated with GSI suffered from weight loss, increased skin cancer and infections [7, 8], in addition to worsening cognition. Similar memory deficits were found using GSIs in AD mice as well as in wild type mice [42, 46]. In treated subjects, pathological changes consistent with inhibition of Notch raised the possibility that this was responsible for treatment failures [7, 8]. Accordingly, an attempt to produce APP specific γ-secretase inhibitors was suggested [43, 44, 91].

The studies reported herein argue that the use of an APP specific GSI will still have deleterious effects on neuronal structure and function. It is possible that a potent GSI used at clinically relevant concentrations, lower than those we employed, may minimize unwanted effects. Given that inhibition of APP processing, the intended purpose of GSIs, can now be shown to result in unwanted and possibly degenerative neuronal changes possibly through accumulation of APP CTFs, even an APP-specific GSI might be toxic. In contrast, the findings for the sGSM tested here showed the ability to reduce the levels of Aβ_42_ and Aβ_40_ without significantly impacting any of the neuronal phenotypes examined, including those for BDNF trafficking and signaling.

GSIs and GSMs both reduce the production of Aβ_42_ and Aβ_40_ but *via* distinct mechanisms [35, 91]. GSIs inhibit production of all Aβ species; GSMs reduce Aβ_42_ and Aβ_40_ and increase the levels of smaller Aβ species, such as Aβ_38_ [34, 36] (also see **Fig. 1A and B**). While the GSI resulted in a marked increase in the level of APP CTFs, no increase was seen with the sGSM (also see **Fig. 1E**). Our studies show that the GSI and sGSM had different effects on a number of neuronal phenotypes. First, the GSI significantly impaired axonal trafficking while the sGSM did not. Second, differences in endosomal transport of BDNF were observed for velocity, net distance, and directionality with the GSI but not the sGSM. Third, changes in Erk1/2 signaling downstream from BDNF were associated with the GSI but not with the sGSM. Since retrograde axonal signaling of BDNF plays a critical role in neuronal maintenance and function [52, 53, 58, 81, 85, 92, 93], we reasoned the GSI would invoke other downstream consequences, including dysregulation of gene expression needed for the maintenance of neuronal circuits. Indeed, the GSI-mediated effect on BDNF-induced Erk1/2 activation and nuclear translocation of pCREB are evidence for this possibility. Further distinguishing the GSI from the sGSM were changes on the structure of organelles in neuronal processes involving both mitochondria and synaptophysin-positive vesicles. In GSI-treated cultures, there was a decrease in density of individual mitochondrial puncta together with accumulation and abutment of mitochondria in processes. The same pattern was seen for synaptic vesicles, a decrease in the apparent density of individual puncta and an increase in their size.

Although it is not clear how changes in neurons were produced by the GSI but not the sGSM, our findings point to a role for APP CTFs. This is supported by differences in APP processing by the GSI and sGSM: While both reduce Aβ species, only the GSI increases APP CTFs levels. In addition, GSI-induced changes were prevented by reducing APP gene expression. Consistently, recent studies showed that altered APP processing, including overexpression of APP CTFs, changed APP trafficking [94] and induced organelle accumulation [95–97] and mitochondrial dysfunction [98–101]. We cannot rule out contributions by γ-secretase substrates, other than APP, in the context of GSI treatment. As one example, GSI-induced changes in dendritic arborization are consistent with earlier reports for inhibition of Notch processing [66, 68, 94].

The most salient outcome of our study is that GSI-mediated disruption of BDNF trafficking and signaling point to GSIs compromising an important neuronal function. Whether or not this played a role in the failure of GSI Phase III clinical trials for treatment of AD [7, 8, 63], or for GSI-induced changes in cognition in animal models is uncertain [42, 46, 70, 71]. But the data suggest that changes in APP processing may create unwanted effects. Nevertheless, in a recently completed large Phase III clinical trial, patients receiving the GSI semagacestat at a lower dose failed to improve cognition. And at a higher dose, functional ability in AD patients became significantly worsening as compared to patients receiving placebo. In addition, severe adverse effects including skin cancers, infections etc were observed with the use of semagacestat in human subjects [8]. Furthermore, in animal studies subchronic dosing with either GSIs or Nortch-sparring GSIs impaired normal cognition in 3-month-old Tg2576 AD mice and even in normal wild type mice[46]. GSIs did not rescue the deficits of memory deficits in a mouse model of familial Danish dementia (FDD) [42]. Taken together, these results strongly suggest that accumulation of APP CTFs, even in the absence of Aβ peptides, caused by GSI treatment, posts significant toxic effects to synaptic function and cognition, among other adverse effects. Accordingly, future attempts to inhibit γ-secretase activity must at the very least examine changes in APP processing and the potential effects of these changes on neuronal function.

In contrast to GSIs, sGSM had little effects on neuronal function in the assays we performed, and could potentially serve as a better therapeutic alternative. This conclusion is supported by other observations pointing to the potential value of GSMs as anti-amyloid based drug therapies [35, 36, 63, 64, 102]. GSMs such as sGSMs[34–36] and EVP-0015962[103] decreases Aβ42 with no apparent effects on either the total Aβ peptides or the level of APP CTFs. Both classes of GSMs have been shown effective in reducing Aβ aggregates, amyloid plaques, inflammatory markers. And most importantly, they have been shown effective in improving cognitive deficits in Tg2576 AD mice[34, 103]. However, given the lessons learned with the GSI endeavors, it is absolutely imperative to carefully examine both *in vitro* and *in vivo* the impact of GSMs on neuronal structure and function prior to their clinical application.

## Supporting Information

S1 Movie QD-BDNF transport in vehicle-treated hippocampal neurons. QD-BDNF movement within the microgroove area of the chamber. Signals that were moving to the right indicate movement in the retrograde direction towards the cell while left-moving signals represent anterograde transport. Signals that showed no movement represent stationary vesicles.
(AVI)

S2 Movie QD-BDNF transport in BMS-299897-treated hippocampal neurons. QD-BDNF movement within the microgroove area of the chamber. As in **1**, signals that were moving to the right indicate movement in the retrograde direction towards the cell while left-moving signals represent anterograde transport. Signals that showed no movement represent stationary vesicles.
(AVI)

S3 Movie QD-BDNF transport in sGSM41-treated hippocampal neurons. QD-BDNF movement within the microgroove area of the chamber. As in **1**, signals that were moving to the right indicate movement in the retrograde direction towards the cell while left-moving signals represent anterograde transport. Signals that showed no movement represent stationary vesicles.
(AVI)

S4 Movie QD-BDNF transport in Vehicle + APP siRNA-treated hippocampal neurons. QD-BDNF movement within the microgroove area of the chamber. As in **1**, signals that were moving to the right indicate movement in the retrograde direction towards the cell while left-moving signals represent anterograde transport. Signals that showed no movement represent stationary vesicles.
(AVI)

S5 Movie QD-BDNF transport in BMS-299897 + ctrl siRNA-treated hippocampal neurons. QD-BDNF movement within the microgroove area of the chamber. As in **1**, signals that were moving to the right indicate movement in the retrograde direction towards the cell while left-moving signals represent anterograde transport. Signals that showed no movement represent stationary vesicles.
(AVI)

S6 Movie QD-BDNF transport in BMS-299897 + APP siRNA-treated hippocampal neurons. QD-BDNF movement within the microgroove area of the chamber. As in **1**, signals that were moving to the right indicate movement in the retrograde direction towards the cell while left-moving signals represent anterograde transport. Signals that showed no movement represent stationary vesicles.
(AVI)
